# Violence outcomes in later adolescence with the Good School Toolkit-Primary: a nonrandomized controlled trial in Uganda

**DOI:** 10.1186/s12889-024-19024-5

**Published:** 2024-06-07

**Authors:** Louise Knight, Lydia Atuhaire, Amiya Bhatia, Elizabeth Allen, Sophie Namy, Katharina Anton-Erxleben, Janet Nakuti, Angel Mirembe, Mastula Nakiboneka, Janet Seeley, Helen A. Weiss, Jenny Parkes, Chris Bonell, Dipak Naker, Karen Devries

**Affiliations:** 1 London School of Hygiene and Tropical Medicine, 15-17 Tavistock Place, London, WC1H 9SH UK; 2 Medical Research Council/Uganda, Virus Research Institute and London School of Hygiene and Tropical Medicine Uganda Research Unit , Plot 51-59 Nakiwogo Road, Entebbe, Uganda; 3 Raising Voices, Plot 16, Tufnell Drive, Kamwokya, Kampala, Uganda; 4 MRC International Statistics and Epidemiology Group, London School of Hygiene & Tropical Medicine, Keppel Street, London, WC1E 7HT UK; 5 UCL Institute of Education, 20 Bedford Way, London, WC1H 0AL UK

**Keywords:** Child abuse, Intimate partner violence, Family connectedness, Whole school intervention, Uganda

## Abstract

**Background:**

We sought to determine whether the Good School Toolkit-Primary violence prevention intervention was associated with reduced victimisation and perpetration of peer and intimate partner violence four years later, and if any associations were moderated by sex and early adolescent: family connectedness, socio-economic status, and experience of violence outside of school.

**Methods:**

Drawing on schools involved in a randomised controlled trial of the intervention, we used a quasi-experimental design to compare violence outcomes between those who received the intervention during our trial (*n* = 1388), and those who did not receive the intervention during or after the trial (*n* = 522). Data were collected in 2014 (mean age 13.4, SD 1.5 years) from participants in 42 schools in Luwero District, Uganda, and 2018/19 from the same participants both in and out of school (mean age 18, SD: 1.77 years). We compared children who received the Good School Toolkit-Primary, a whole school violence prevention intervention, during a randomised controlled trial, to those who did not receive the intervention during or after the trial. Outcomes were measured using items adapted from the International Society for the Prevention of Child Abuse and Neglect Child Abuse Screening Tool-Child Institutional. We used mixed-effect multivariable logistic regression, with school fitted as a random-effect to account for clustering.

**Results:**

1910 adolescents aged about 16–19 years old were included in our analysis. We found no evidence of an average long-term intervention effect on our primary outcome, peer violence victimization at follow-up (aOR = 0.81, 95%CI = 0.59–1.11); or for any secondary outcome. However, exposure to the intervention was associated with: later reductions in peer violence, for adolescents with high family connectedness (aOR = 0.70, 95% CI 0.49 to 0.99), but not for those with low family connectedness (aOR = 1.07, 95% CI 0.69 to 1.6; p-interaction = 0.06); and reduced later intimate partner violence perpetration among males with high socio-economic status (aOR = 0.32, 95%CI 0.11 to 0.90), but not low socio-economic status (aOR = 1.01 95%CI 0.37 to 2.76, p-interaction = 0.05).

**Conclusions:**

Young adolescents in connected families and with higher socio-economic status may be better equipped to transfer violence prevention skills from primary school to new relationships as they get older.

**Trial registration:**

Clinicaltrials.gov, NCT01678846, registration date 24 August 2012.

Protocol for this paper: https://www.researchprotocols.org/2020/12/e20940.

**Supplementary Information:**

The online version contains supplementary material available at 10.1186/s12889-024-19024-5.

## Introduction

Globally, over one billion children experience violence every year [1, 2] and one in three women have experienced physical and/or sexual intimate partner violence, or sexual violence by a non-partner in their lifetime [3–5], leading to adverse health consequences [6]. A large body of evidence shows that experiencing and perpetrating violence in childhood is associated with violence victimization [7–10] and violence perpetration [7] later in adolescence and in adulthood, indicating that prevention efforts must begin at an early age [4, 11].

Several promising interventions suggest that peer violence, [12] bullying, [13, 14] and dating violence [15] can be reduced through school-based approaches. Evidence from school-based interventions in high-income countries show reductions in violence, bullying, and dating violence three to five years later [13, 15–19]. In most of these studies long term outcomes were assessed while children were still in the same school, [13, 16–19] with a limited number of studies measuring long term effects outside of school settings [15]. Few studies examined the role of home or community environments in the effectiveness of school-based interventions, especially over the longer term, after adolescents transition out of schools where interventions have been implemented. There is no evidence from low- or middle-income countries. In this paper, we explore whether the Good Schools Toolkit-Primary intervention (referred to as ‘the Toolkit’ herein) had long-term effects on peer and intimate partner violence (IPV) victimization and perpetration among children exposed to the intervention during their time in primary school in Uganda.

### Objectives

In this paper, we tested three a-priori hypotheses [22]. First, we sought to examine whether exposure to the Toolkit was associated with reduced peer violence victimization four years later (primary outcome). Second, we sought to explore whether exposure to the intervention was associated with reductions in the following secondary outcomes, four years later: (a) peer violence perpetration; (b) intimate partner violence victimization among ever partnered female adolescents, and (c) intimate partner violence perpetration among ever partnered male adolescents. Our third objective was to examine whether any reductions in violence perpetration and victimization four years later would vary across four moderators. One, we hypothesised effects would be smaller among boys, as norms encouraging the use of violence are highly bound with masculinity in this context. Two, we hypothesised smaller effects among adolescents who had, at primary school: low socioeconomic status, lower levels of family connectedness, and who had experienced violence outside of the school setting from caregivers, community members or others. Poor, less connected adolescents or those who had experienced more violence were thought to have structural environments where they would be confronted with more violence and have more difficulty putting into practice learnings from the Toolkit.

## Methods

### Study design

We designed a nonrandomized quasi-experimental study, taking advantage of the variation in delivery of the Toolkit during and after the GSS trial. The design of the GSS trial is described elsewhere [21, 23]. In the GSS trial, we selected a stratified random sample of 42 schools from Luwero district, Uganda. Luwero district is adjacent to Kampala, the capital of Uganda, and is demographically similar to Uganda as a whole. Uganda is classified as one of the least developed countries in the world, according to the World Bank. It also has a supportive policy environment for violence prevention, and is a pathfinder country in the Global Partnership to End Violence Against Children.

All schools approached to participate agreed. Stratified randomization was used to allocate schools to receive the intervention immediately or to be wait-listed to receive the intervention after the end of the trial.

### Intervention

The Toolkit is a violence prevention intervention developed by and freely available from the Ugandan non-profit organisation Raising Voices (www.raisingvoices.org). It is a complex whole-school intervention that draws on the Transtheoretical Model [20] to improve school operational culture and prevent teacher and peer violence. The intervention is school-led through two appointed teacher and student protagonists. Materials are provided along with over 60 activities that engage the whole school as they sequentially complete six core steps. During 2012–2014, as part of the Good School Study (GSS; referred to as the ‘GSS trial’ herein), we found that exposure to the Toolkit intervention reduced past week staff-to-student physical violence (primary outcome), [21] staff-to-student emotional violence, and any student-to-student past-week physical, emotional and/or sexual violence [12, 26]. The Toolkit intervention was originally designed for primary schools, but Raising Voices hypothesised that the effects of exposure to the Toolkit would ‘travel with’ adolescents as they aged [22]. In particular, Toolkit learnings for children around power, behaviour management and relationship building could plausibly result in less violence in future relationships both inside and outside the school context, including both peer and intimate partnerships.

### Participants and study procedures

**Fig. 1:**
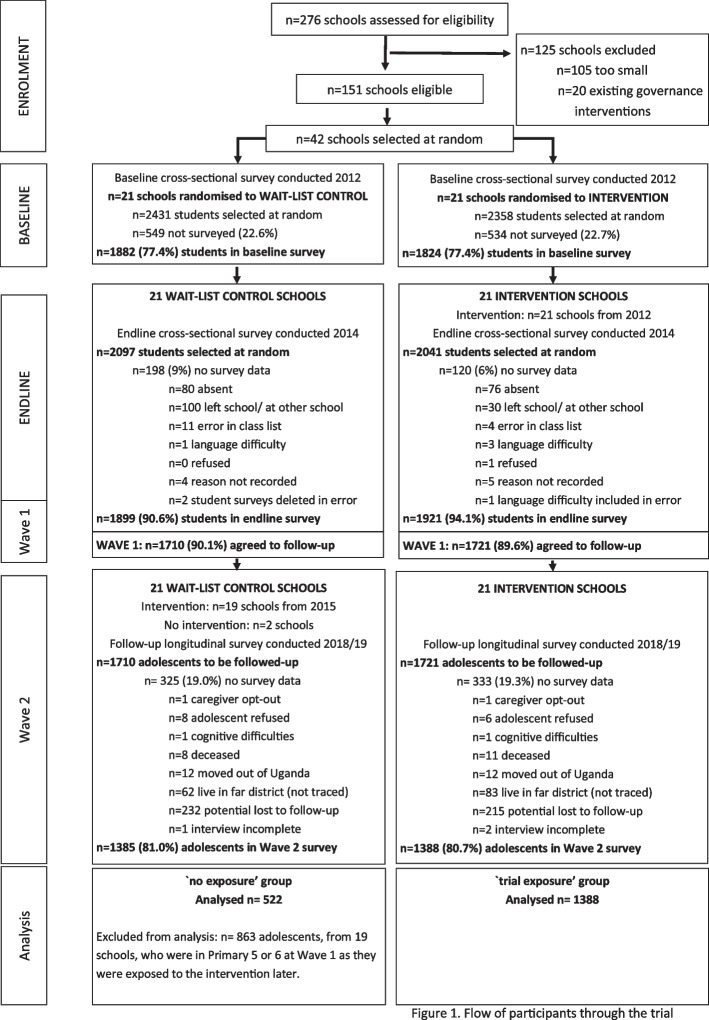
Flow of participants through the trial

Full details of sampling, recruitment, consent and referral procedures are published elsewhere for wave 1 [26] and wave 2 [24]. Briefly, our wave 1 sample is comprised of 3431 adolescents aged 11–14 years who participated in the endline survey of the GSS trial in 2014, and agreed to be followed up (corresponding to 90% of the full trial endline survey sample) [24]. Wave 2 data were collected from 81% (2773/3431) of the wave 1 sample, between October 2018 and August 2019. In this paper, we use data from all 1910 adolescents aged 16–19 years who completed the wave 1 and wave 2 surveys and were either exposed to the intervention during the trial or were not exposed to the intervention (Fig. 1 Flow of participants through the trial). Although all of the participants took part in our original randomised controlled trial, we consider the comparison between trial exposure and no exposure groups non-randomised because eligibility for the no exposure group differs systematically from eligibility to the trial exposure group, and allocation to these groups was not random.

At wave 2, adolescents age 18 years old or more, and emancipated minors, provided informed written consent prior to participation. For adolescents under 18 years old, who were not emancipated minors, caregivers were provided with information and could opt-out their child from participating. Adolescents were approached to provide informed written assent, and all participants’ ability to understand consent procedures in English or Luganda was assessed before they were invited to participate. Interviewers who received extensive training on violence data collection administered face-to-face survey interviews in Luganda or English and recorded responses on hand-held devices. Female interviewers interviewed both male and female participants; male interviewers interviewed only male participants. Where two cohort participants were in an intimate relationship, both partners were interviewed only if it was safe to do so. All data were recorded, transmitted and stored on a secure server using Open Data Kit (ODK). Referral to child protection or other services was based on predefined criteria agreed with service providers that related to the severity and timing of violence and/or mental health concerns reported. All adolescents were offered counselling regardless of what they disclosed.

### Outcome measures

All outcomes were measured at wave 2, were self-reports, and were constructed as binary outcomes. Question items are detailed in Annex Table 1. The primary outcome was any emotional, physical and/or sexual violence victimisation from a peer in the last year, measured using questions adapted from International Society for the Prevention of Child Abuse and Neglect Child Abuse Screening Tool-Child Institutional (ICAST-CI) [25]. A peer was defined as someone of a similar age, and could include friends, students, co-workers or community members. We examined three secondary outcomes: (a) peer violence perpetration, using items adapted from the ICAST-CI, (b) intimate partner violence (IPV) victimisation among female adolescents, and (c) IPV perpetration among male adolescents. An intimate partner was defined as a boyfriend or girlfriend, husband or wife or casual dating partner, of any age. IPV questions were adapted from the WHO Multi-Country study on women’s health and domestic violence against women, [26] and the Conflict in Adolescent Dating Relationships Inventory [27, 28].

### Moderators

We included four variables identified apriori [22] as possible moderators, all of which were all measured at wave 1. First, family connectedness was measured using four question items (I feel: 1) like my parents/caregivers care about me, 2) safe at home, 3) like I belong at home, and 4) I like to spend time at home) [29]. Likert-type responses were coded 0–3, a score calculated (range 0 to 12; Cronbach alpha of 0.70) and grouped into high or low at the median. Second, we included male or female sex of the adolescent. Third, socioeconomic status was proxied by the number of meals eaten yesterday and, as for previous analysis, was included as a binary variable (< 3 meals or 3 + meals) [30, 31]. Fourth, any type of violence ever experienced outside of school from any perpetrator (other than school staff or other students) was included as a binary variable.

### Toolkit exposure measures

The ‘trial exposure’ group includes Primary 5 to 7 students who attended one of the 21 intervention schools. The ‘no exposure’ group includes both: Primary 7 students from the 19 wait-list control schools that implemented the intervention, as they would have left school prior to post-trial intervention delivery; and Primary 5 to 7 students who had attended 2 of the 21 wait-list control schools who declined to implement the intervention after the GSS trial (Fig. 1).

### Power to detect a difference

We carried out an indicative power calculation using a two sided, two sample proportion test based on the following assumptions: 2350 pupils were available to follow up; 50% of our adolescent sample would report past year experience of physical, emotional and/or sexual violence from a peer in our ‘no exposure’ groups; [32] the ratio of exposed to not exposed was 3:1; and 72% of the exposed were followed up giving a sample size of 1700. Based on these the smallest difference we could detect between `no exposure’ and `trial exposure’ groups, with alpha of 5% and power of 80%, is an 8-percentage point absolute difference. No ICC was used in these indicative calculations as the allocation to exposure and no exposure groups was not by cluster.

### Statistical analysis

For descriptive analysis, we estimated means and standard deviations (SDs) for normally distributed data, and medians and ranges or interquartile ranges (IQRs) for non-normally distributed variables. We formally tested for differences in characteristics across exposure groups (Table 2), and survey completion across waves (Annex: Table 1). All descriptive analyses accounted for clustering by primary school (by using the Stata *Svy* command), using Taylor linearized variance estimation to calculate standard errors and corrected Pearson Chi-squared statistics for categorical data. For those with endline Wave 2 data, missing data was less than 1% missing for any variable analysed.

Our primary analysis compared binary violence outcomes in the ‘trial exposure’ versus `no exposure’ groups. We fit multivariable mixed-effect logistic regression to estimate odds ratios and 95% confidence intervals (CIs), adjusting for clustering by including school as a random effect [33]. All models include school-level mean outcomes collected at baseline, prior to the start of the GSS trial [21, 34]. Adjusted models include sex, number of meals eaten yesterday, and primary school grade, because these factors were associated with non-completion of a Wave 2 survey (Annex Table 2), as well as family connectedness and experience of violence outside of school. Primary school grade was also associated with both exposure grouping and violence outcomes.

We then conducted moderation analyses to examine pre-specified subgroup effects by: family connectedness, sex, number of meals eaten yesterday, and experience of violence outside of school, measured at wave 1. Due to multiple testing and the likelihood of type 1 error, the size and direction of effects and confidence intervals were considered when assessing results and patterns across outcomes [35]. Moderation was assessed by comparing fully adjusted models fitted with and without an interaction term. Likelihood ratio tests with *p*-values < 0.1, along with the direction and size of effect for each group, were considered as suggestive evidence for moderation. Where there was evidence of moderation, stratum-specific subgroup effects were calculated directly from the adjusted model. All analyses were conducted using Stata 15.0.

### Ethical review of study

This study received ethical approval from LSHTM (6183 and 14,768), University of London (UCL), Institute of Education (IoE), Research Ethics Committee (1091), Uganda Virus Research Institute (UVRI) and Uganda National Council of Science and Technology (UNCST) ethics committees (SS2520 and SS4722).

## Results

### Sample characteristics

1910 adolescents were included in the present analysis (Fig. 1). At Wave 1, the mean age was 13 years, female adolescents represented 53% or more of sample in each group, less than half of the adolescents reported eating three or more meals yesterday, and 27% (trial exposure) and 30% (no exposure) of children reported an experience of violence outside the school. Four years later, at wave 2, the mean age of adolescents was 18 years (SD: 1.77 years). Wave 1 adolescent characteristics were similar across the trial exposure and no exposure groups (Table 1), and attrition between the Wave 1 and 2 surveys was similar between the groups (Fig. 1).

**Table 1:** Wave 1 characteristics by Good School Toolkit-Primary intervention exposure groups

	Trial exposure		No exposure		
Number of schools and school grades	21 schools		2 schools P5-P7		
	P5-P7		19 schools P7		
	n/N %		n/N %		*P*-value^a^
Number in group/total Wave 2 survey	1388/2773	50	522/2773	19	-
Follow-up rate^b^	1388/1721	81	522/634	82	-
Wave 1 characteristics:					
Age in years, mean (SD)	13.06	1.54	13.44	1.55	0.27
Sex					
Male	648/1388	47	231/522	44	0.47
Female	740/1388	53	291/522	56	
Meals eaten yesterday					
One meal or less	243/1387	18	55/521	11	0.11
Two meals	559/1387	40	209/521	40	
Three or more meals	585/1387	42	257/521	49	
Family connectedness score, mean (SE)^c^	9.99	0.11	10.01	0.08	0.93
Any violence outside of school, ever	373/1388	27	159/522	30	0.41

^a^*P*-value: linearised SE with corrected person *Chi2p*-value

^b^proportion completed Wave 2 survey/ total completed Wave 1 survey (within each exposure group)

^c^Family connectedness score *n* = 1906

### Is Good School Toolkit-Primary school exposure associated with long-term violence reductions?

**Fig. 2:**
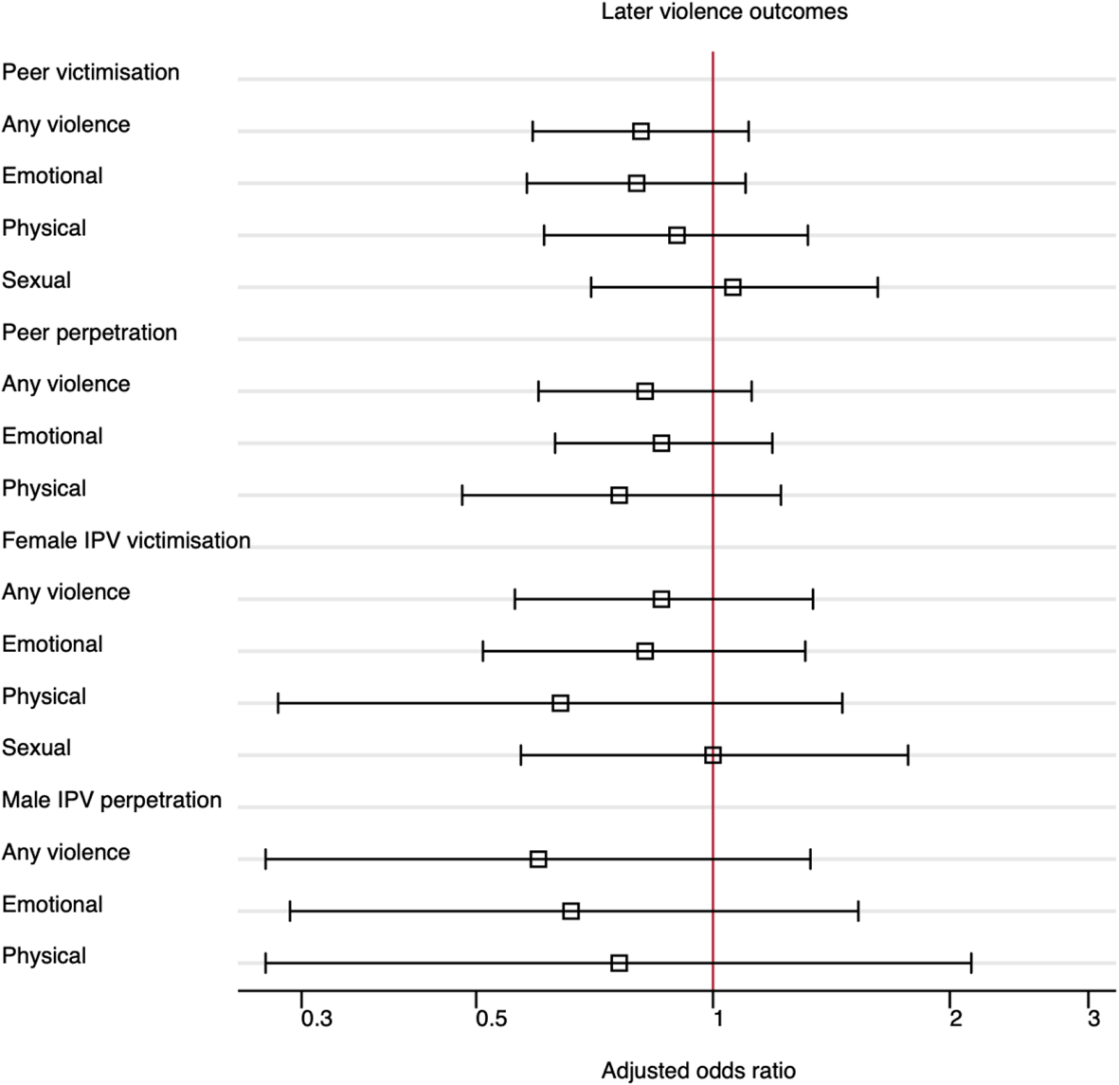
Effect of the Toolkit on pre-specified outcomes of interest. Adjusted odds ratios below 1 favor the intervention, adjusted odds rations above 1 favor control. Square boxes are point estimates and bars are 95% confidence intervals

Peer violence victimisation at wave 2 was reported by 61% of adolescents in the `trial exposure’ compared to 64% in the `no exposure’ group (adjusted odds ratio, aOR = 0.81; 95% confidence interval, 95%CI 0.59 to 1.11; Table 2). There was also no evidence of a difference between the `trial exposure’ and `no exposure’ groups for any secondary outcome (Table 2, Fig. 2).

**Table 2:** Good School Toolkit-Primary intervention effect on later violence outcomes

	Trial exposure	No exposure	Trial vs no exposure	
	n (%)	n (%)	Basic OR [95% CI]	Adjusted^a^ OR [95% CI]
Peer violence victimisation^b^, denominator	1388	522	1910	1904
**Primary outcome:**				
Any violence, past year	842 (61)	335 (64)	0.84 [0.63,1.12]	0.81 [0.59,1.11]
**Secondary outcomes:**				
Emotional violence, past year	783 (56)	317 (61)	0.82 [0.62,1.09]	0.80 [0.59,1.10]
Physical violence, past year	235 (17)	78 (15)	1.16 [0.84,1.61]	0.91 [0.62,1.33]
Sexual violence, past year	203 (15)	61 (12)	1.17 [0.81,1.68]	1.07 [0.71,1.62]
Peer violence perpetration^c^, denominator	1388	522	1910	1904
Any violence, past year	400 (29)	153 (29)	0.87 [0.66,1.15]	0.83 [0.60,1.14]
Emotional violence, past year	332 (24)	127 (24)	0.88 [0.66,1.17]	0.88 [0.64,1.22]
Physical violence, past year	166 (12)	55 (11)	0.97 [0.67,1.42]	0.76 [0.47,1.21]
Female IPV victimisation^b^, ever partnered^e^, denominator	401	161	562	560
Any violence, past year	218 (54)	87 (54)	1.09 [0.75,1.58]	0.86 [0.55,1.36]
Emotional violence, past year	165 (41)	67 (42)	1.00 [0.67,1.47]	0.82 [0.50,1.33]
Physical violence, past year	37 (9)	13 (8)	1.10 [0.56,2.14]	0.65 [0.28,1.50]
Sexual violence, past year	122 (30)	41 (25)	1.21 [0.75,1.96]	0.99 [0.56,1.76]
Male IPV perpetration^d^, ever partnered^e^, denominator	354	143	497	495
Any violence, past year	60 (17)	35 (24)	0.68 [0.33,1.41]	0.61 [0.27,1.33]
Emotional violence, past year	53 (15)	24 (17)	0.76 [0.36,1.60]	0.68 [0.29,1.62]
Physical violence, past year	17 (5)	10 (7)	0.66 [0.26,1.67]	0.77 [0.27,2.18]

^a^All adjusted models include sex, number of meals eaten yesterday, primary school grade, family connectedness and violence outside of school at Wave 1

Basic and adjusted models include school-level baseline mean of ^b^ specific type of peer violence victimisation outcome, ^c^physical violence perpetration towards anyone, and ^d^physical and/or sexual violence perpetration towards anyone

^e^Ever partnered defined as ever having a boy/girlfriend, husband/wife, or causal dating partner

### Do any characteristics of adolescents moderate the effects of exposure to the Toolkit on long-term violence reductions?

We found some evidence of moderation in line with our a priori hypotheses for family connectedness (Table 3), suggesting that `trial exposure’ compared to `no exposure’ was associated with reductions in later any peer violence victimization among adolescents reporting high family connectedness at the Wave 1 survey compared to those reporting low family connectedness. We also found evidence that male adolescents in the ‘trial exposure’ group versus the ‘no exposure’ group were less likely to report intimate partner violence perpetration at wave 2 if they had eaten 3 meals or more the day before the Wave 1 survey, compared to those who had not eaten at least 3 meals. No other moderators were associated with any differential effects of Toolkit exposure, although associations were in expected directions (Analysis provided on request).

**Table 3:** Moderation of Good School Toolkit-Primary intervention effect on later peer violence outcomes in the past year

Moderator	Family connectedness			Violence outside of school			Number of meals, past day			Sex		
	aOR [95% CI]	aOR [95% CI]	p	aOR [95% CI]	aOR [95% CI]	p	aOR [95% CI]	aOR [95% CI]	p	aOR [95% CI]	aOR [95% CI]	p
Subgroup	Low	High		None	Violence		< 3 meals	3 + meals		Male	Female	
Total n/N, % in subgroup	644/1904, 34%	1262/1904, 66%		1378/1904, 72%	532/1904, 28%		1066/1904, 56%	842/1904, 44%		882/1910, 46%	1028/1910, 54%	
**Primary outcome**												
Any peer victimisation	1.08 [0.70,1.67]	0.70 [0.49,0.99]	0.06	0.78 [0.55,1.09]	0.90 [0.56,1.43]	0.55	0.82 [0.55,1.21]	0.80 [0.55,1.17]	0.95	0.93 [0.63,1.37]	0.72 [0.50,1.05]	0.25
**Secondary outcomes**												
Any peer perpetration	1.06 [0.67,1.66]	0.73 [0.52,1.04]	0.14	0.79 [0.56,1.12]	0.93 [0.58,1.40]	0.51	0.85 [0.58,1.25]	0.80 [0.54,1.20]	0.81	0.75 [0.50,1.11]	0.91 [0.62,1.34]	0.39
Total n/N, % in subgroup	181/560, 32%	381/560, 68%		368/560, 65%	194/560, 35%		345/560, 62%	215/560, 38%				
Female IPV victimisation	0.86[0.42,1.77]	0.86[0.51,1.44]	0.99	0.83 [0.48,1.43]	0.91 [0.47,1.78]	0.82	0.79 [0.46,1.37]	0.98 [0.51,1.87]	0.59			
Total n/N, % in subgroup	165/495, 33%	330/495, 67%		373/495, 75%	124/495, 25%		281/495, 57%	216/495, 50%				
Male IPV perpetration	0.75 [0.24,2.39]	0.56 [0.23,1.36]	0.61	0.51 [0.21,1.25]	0.92 [0.29,2.92]	0.32	1.02 [0.37,2.84]	0.33 [0.12,0.96]	0.06			

All adjusted models include sex, number of meals eaten yesterday, primary school grade, family connectedness and violence outside of school at Wave 1 (if not subgroup being explored). No outcome *n* < 10 in any group. *P* values are from Likelihood Ratio tests

*aOR* Adjusted odds ratio; effect of Good School Toolkit trial exposure versus no exposure on primary and secondary outcomes

## Discussion

In this nonrandomised trial, we did not find evidence of an average effect of Toolkit exposure on peer or intimate partner violence 4 years later, after adolescents had transitioned out of intervention primary schools. However, we did find evidence that the Toolkit reduced later peer violence victimisation for adolescents who come from families with high connectedness, but not among adolescents who come from families with low connectedness. We also found evidence that the Toolkit reduced later intimate partner violence perpetration among males with higher socio-economic status, but not among those with lower socio-economic status. There was no other consistent evidence of any differential effects by subgroup.

In general, further work is required to understand how the positive effects of school-based violence prevention interventions may be sustained over the long term and transferred to new relationship contexts. The Toolkit is a whole-school intervention, not directly tackling intimate partner violence or sexual violence, but aiming to enhance capacities that may improve multiple violence and other outcomes as adolescents get older. It might be that more specific strategies for violence prevention in relationships encountered in older adolescence are needed, rather than general skill building. Other researchers suggest that multi-setting interventions across schools, homes and communities might be most effective; however, these may be costly [38]. Research in high-income settings highlights the potential of interventions that modify the whole-school environment to impact on a broad range of outcomes among young people [13], and our findings support this.

Our findings do suggest that supportive environments both within and beyond schools could be needed to enable children to sustain positive effects of school-based interventions on violence victimization and perpetration later in life. Other studies have also identified adolescent family connectedness as an important protective factor for violence in adulthood, including intimate partner violence [36]. Families with more supportive environments are likely to have afforded adolescents more opportunities to practice and reflect on new behaviours. It is also likely that structural factors, such as poverty and labour migration, may contribute to adverse home conditions including low family connectedness. Children in these conditions may have been less likely to attend school and therefore may have been less exposed to the intervention.

Our results also show that exposure to the Toolkit is associated with reduced intimate partner violence perpetration by young men of higher socio-economic status, but not among those of lower socio-economic status. Qualitative studies on masculinities and violence suggest that participation in and use of violence as an alternate route to attaining or maintaining a masculine identity can be important for men of lower socio-economic status, who may have less access to income, jobs, and connections with which to establish and practice their masculinities [37]. While young men of higher status may have already been on their way to using less violence with intimate partners, the Toolkit seems to have enhanced this trajectory further. Further research is needed to understand why—it could be that the Toolkit provided less violent social contexts for these young men, thereby changing norms around the acceptability of violence; or similar to family connectedness above, having more resources at home and thus a less stressful environment could have enabled young men more opportunity to practice the use of new behaviours.

### Strengths and limitations

Our four year follow up period allowed us to examine whether intervention effects persist as adolescents get older and have left intervention schools. Our cohort is broadly representative of adolescents in Luwero District, and was not selected based on any characteristics related to the intervention [21]. We find a low attrition and high response rate. However, Uganda, and Luwero, may differ from other contexts in important ways and it is not clear that our findings would generalise to other settings.

Although strict study procedures were followed to ensure participants were interviewed in an environment where they felt safe to disclose experiences, it is likely that levels of violence were underestimated. Some of our measures also have limitations, such as number of meals per day, which may not fully capture variation in socio-economic status, and family connectedness, which contains one item related to feelings of safety at home and thus could plausibly be capturing aspects of home experience related to violence.

Our quasi-experimental design uses an unexposed comparison group that arises from the timing of later delivery to school grades. To address differences across comparison groups, efforts have been made to control for confounding by school grade in analysis, and we also consider consistency across findings when interpreting results [21]. We adjust for other confounders measured at Wave 1, which means they are not associated with intervention exposure; however this also may mean that the values of these confounding variables may have changed over the four years between waves of data collection. We may have been underpowered to assess differential intervention effects for subgroups, and have also conducted multiple tests [39]. We thus considered patterns when interpreting results.

### Future directions

Our findings suggest that enhanced or additional intervention ‘boosters’ could be evaluated to see if they effectively support long-term positive change for different subgroups of students. Examining whether and how secondary school interventions can support gains made at primary school is of particular interest.

Our findings also underscore the importance of considering family context and connectedness in research on school-based interventions. Family environment—both psychological and economic—may be a key area of additional support for school interventions to avoid widening inequalities in violence and other health outcomes. Future directions for intervention might include developing and testing complementary intervention packages that engage both schools and caregivers (for example, via parenting interventions, and/or cash transfer programmes). Economic strengthening interventions at a macro-level may also help to bolster the effects of violence prevention programmes. Further research is needed on how to further tailor interventions to the needs and realities of children with limited resources and support at home.

## Conclusions

The Good School Toolkit-Primary intervention is associated with large reductions in teacher and peer violence among children in primary schools, immediately after 18 months of implementation. When those children transition out of primary school, we did not find clear evidence of reduced violence in peer and intimate partner relationships four years later for all children. However, children who had more connected families and families with higher socio-economic status while they were receiving the primary school intervention did have better violence outcomes four years later. Those with more supportive and better off families may be better able to carry forward new attitudes and practices from school-based violence prevention interventions.

### Supplementary Information

Supplementary Material 1.

## Data Availability

No datasets were generated or analysed during the current study.
