# Supplementary material for: Violence outcomes in later adolescence with the Good School Toolkit-Primary: a nonrandomized controlled trial in Uganda
Source: BMC Public Health. 2024 Jun 7;24:1532. doi: 10.1186/s12889-024-19024-5 (PMC11157797; doi:10.1186/s12889-024-19024-5)
Supplement: Supplementary file 1 — Supplementary Material 1. [file 12889_2024_19024_MOESM1_ESM.docx]

**Annex**

Table A1: Description of violence questions and outcomes measures

| **Measures** | **Questions** | **Measure construct** |
| --- | --- | --- |
| **Peer violence** | A peer was defined as someone of a similar age who might be a friend, another student at school, a co-worker of similar age or someone you know from your community of a similar age. |  |
| **Victimisation:**  **(primary outcome)**  Self-reported experience of any physical, emotional and/or sexual violence from a peer, in the last 12 months. | Emotional   1. Insulted you, cursed you, or called you rude or hurtful names? 2. Tried to embarrass or humiliate you? 3. Referred to your skin colour/ gender/ religion/ tribe or health problems you have in a hurtful way? 4. Refused to speak with you, sit with you or did something else to make you feel bad or lonely? 5. Spread rumours about you or tried to turn your friends against you? 6. stole or broke or ruined your belongings?   Physical   1. Slapped you with a hand on your face, head or body? 2. Pushed or shoved you very hard to hurt you? 3. Punched you or hit you with a closed fist? 4. Severely beat you up?   Sexual   1. Disturbed or bothered you by making sexual comments about you? 2. Kissed you, when you did not want them to? 3. Give you money/ things to do sexual things? 4. Touched your genitals or breasts when you did not want them to, or in a way that made you uncomfortable? 5. Threatened or pressured you to make you do something sexual with them? 6. Had sex with you, by physically forcing you? | Question items on physical (6 items), emotional (4 items) and sexual violence (4 items) experienced from a peer, constructed as binary outcome. Positive response to one or more of the 16 items coded=1, else coded=0.  Binary outcomes also constructed separately by violence type (physical, emotional and sexual). |
| **Perpetration:**  Self-reported use of physical and/or emotional violence against a peer, in the last 12 months. | Emotional:   1. Insulted, cursed, or called a peer a rude or hurtful names? 2. Tried to embarrass or humiliate a peer? 3. spread rumours about a peer or tried to turn their friends against them?   Physical:   1. Slapped a peer with your hand on their face, head or body? 2. Punched a peer with a closed fist? | Questions items on physical (2 items) and emotional violence (3 items) use against a peer, constructed as binary outcome. Positive response to one or more of the 5 items coded=1, else coded=0.  Binary outcomes also constructed separately by violence type (physical and emotional) |
| **Intimate Partner Violence (IPV)** | An intimate partner was defined as: boyfriend/girlfriend, married, or causally dating. |  |
| **Victimization, female:**  Self-reported experience of physical, emotional and/or sexual IPV among ever partnered adolescent women, in the last 12 months. | Emotional:   1. Spread rumours about you or tried to turn your friends against you? 2. Spoken to you in a mean (hostile) tone of voice? 3. Insulted you or deliberately said things to make you feel bad about yourself? 4. Made fun of or humiliated you in front of other people? 5. Taken your money or any of your things against your will? 6. Tried to scare or intimidate you on purpose, for example by the way they you looked at you, by shouting, or by smashing things? 7. Threated to hurt or actually hurt people you care about as a way of hurting you, or damage things of importance to you? 8. Threatened to hurt you for example by hitting you, throwing something at you or using a weapon against you?   Physical:   1. Twisted your arm or any other body part, slapped you, pushed you, pulled your hair or thrown something at you? 2. Hit you with a fist or something else that could hurt you, kicked, dragged, or beat you up? 3. Choked you, burnt you, forced you to do something that was dangerous, or used a knife, panga, gun or other weapon against you?   Sexual:   1. Kissed or touched you sexually when you did not want them to? 2. Pressured you to do sexual things in return for money, gifts or favours? 3. Did you ever have sex with a partner when you did not want to, because you were afraid of what they might do? 4. Forced you to have sex with them when you did not want to? | Question items on physical (3 items), emotional (8 items) and sexual violence (4 items) experienced from a partner, constructed as a binary outcome. Positive response to one or more items coded=1, else coded=0.  Binary outcomes also constructed separately by violence type (physical, emotional and sexual). |
| **Perpetration, male:**  Self-reported use of physical and/or emotional IPV, among ever partnered adolescent men, in the last 12 months. | Emotional:   1. Insulted, cursed, or called a partner a rude or hurtful names? 2. Tried to embarrass or humiliate a partner? 3. Spread rumours about a partner or tried to turn a friends against a partner?   Physical:   1. Slapped a partner with a hand on their face, head or body? 2. Punched a partner or hit them with a closed fist? | Question items measuring physical (2 items) and emotional (3 items) violence perpetration constructed as a binary outcome. Positive response to one or more items coded=1, else coded=0.  Binary outcomes also constructed separately by violence type (physical and emotional). |

Table A2: Wave 1 characteristics compared across Wave 1 and Wave 2 survey completion

| Wave 1 characteristics | Wave 1 | | Wave 1 and Wave 2 survey | | Wave 1  survey only | | P-value^1^ |
| --- | --- | --- | --- | --- | --- | --- | --- |
| Total, % | 3431 | | 2773/3431 81% | | 658/3431 19% | | - |
| Age (years), mean(SE) | 13.02 | (0.09) | 12.98 | (0.09) | 13.17 | (0.09) | 0.004 |
| Sex |  |  |  |  |  |  |  |
| Male | 1578/3431 | 46% | 1322/2773 | 48% | 256/658 | 39% | <0.0001 |
| Female | 1853/3431 | 54% | 1451/2773 | 52% | 402/658 | 61% |  |
| Primary school grade |  |  |  |  |  |  |  |
| Primary 5 | 1302/3431 | 38% | 1026/2773 | 37% | 276/658 | 42% | 0.03 |
| Primary 6 | 1176/3431 | 34% | 950/2773 | 34% | 226/658 | 34% |  |
| Primary 7 | 953/3431 | 28% | 797/2773 | 29% | 156/658 | 24% |  |
| Meals eaten yesterday |  |  |  |  |  |  |  |
| One meal or less | 542/3428 | 16% | 415/2770 | 15% | 127/658 | 19% | 0.02 |
| Two meals | 1356/3428 | 40% | 1092/2770 | 39% | 264/658 | 40% |  |
| Three or more meals | 1530/3428 | 45% | 1263/2770 | 46% | 267/658 | 41% |  |
| Study arm |  |  |  |  |  |  |  |
| Trial intervention | 1721/3431 | 50% | 1388/2773 | 50% | 333/658 | 51% | 0.86 |
| Wait-list control | 1710/3431 | 50% | 1385/2773 | 50% | 325/658 | 49% |  |

^1^ p-value: Wave 1 and 2 survey completed compared to Wave 1 survey only, linearised SE are presented with corrected person *Chi2* p-value
